# Draft Genome Sequences of Azole-Resistant and Azole-Susceptible Aspergillus turcosus Clinical Isolates Recovered from Bronchoalveolar Lavage Fluid Samples

**DOI:** 10.1128/MRA.01446-18

**Published:** 2019-01-03

**Authors:** Maxime Parent-Michaud, Philippe J. Dufresne, Éric Fournier, Christine Martineau, Sandrine Moreira, Vincent Perkins, Louis de Repentigny, Simon F. Dufresne

**Affiliations:** a Department of Microbiology, Infectious Diseases and Immunology, Université de Montréal, Montréal, Québec, Canada; b Laboratoire de santé publique du Québec, Institut national de santé publique du Québec, Sainte-Anne-de-Bellevue, Québec, Canada; c Department of Food Sciences and Nutrition, Université Laval, Québec City, Québec, Canada; d Division of Infectious Diseases and Clinical Microbiology, Department of Medicine, Maisonneuve-Rosemont Hospital, Montréal, Québec, Canada; Broad Institute of MIT and Harvard University

## Abstract

We present the draft genome sequences of two clinical strains of Aspergillus turcosus, one azole-susceptible (strain HMR-AF-23/LSPQ-01275) and the other azole-resistant (strain HMR-AF-1038/LSPQ-01280), isolated from bronchoalveolar lavage fluid of two adult patients. These two strains are the first reported clinical isolates of A. turcosus.

## ANNOUNCEMENT

Invasive aspergillosis is a serious fungal infection of major concern in immunocompromised patients and is associated with a high mortality rate (1–4). Aspergillus fumigatus is the most frequent species causing invasive aspergillosis. Its genome was completely sequenced in 2005, along with A. nidulans and A. oryzae (5–7). *Aspergillus* section *Fumigati* comprises about 60 species (8), often misidentified as A. fumigatus by morphotyping (9–12). First described in 2008, Aspergillus turcosus is a rare species belonging to this section (13). The two isolates whose complete genomes are presented herein are the first reported clinical isolates and the first occurrence of this species outside South Korea.

Four A. turcosus were identified among a collection of 1,186 presumed A. fumigatus clinical isolates collected at Maisonneuve-Rosemont Hospital in Montréal, Québec, Canada. Identification was based on phenotypic characteristics and confirmed by sequencing the following three relevant loci: beta-tubulin, calmodulin, and internal transcribed spacer (13, 14). Antifungal susceptibility testing, performed according to the Clinical and Laboratory Standards Institute (CLSI) broth microdilution procedure (15), revealed reduced susceptibility of strain HMR-AF-1038 to voriconazole, itraconazole, and posaconazole, compared to wild-type A. fumigatus (16). We sequenced its whole genome, along with that of a randomly selected azole-susceptible A. turcosus isolate (HMR-AF-23), for comparison. HMR-AF-23 and HMR-AF-1038 were isolated from bronchoalveolar lavage fluid samples in 2000 and 2012, respectively.

Whole-genome sequencing was performed at the provincial public health laboratory. Isolates were grown on potato dextrose agar at 30°C for 4 to 7 days, then DNA was extracted from conidia with the ZR fungal/bacterial DNA miniprep kit (Zymo Research Corp.). The Nextera XT library preparation kit (Illumina, Inc.) was used to prepare sequencing libraries for both strains. They were then sequenced with a MiSeq 600-cycle sequencing kit v3 as paired-end reads (2 × 300 bp) on a MiSeq sequencing platform (Illumina). HMR-AF-23 generated 25,658,892 raw reads, and the mean per-base sequence depth reached 85×. HMR-AF-1038 generated 25,586,278 raw reads for a 42× per-base mean coverage. Reads were quality and quantity controlled with FastQC version 0.11.5 (https://www.bioinformatics.babraham.ac.uk/projects/fastqc/) and then cleaned with Trimmomatic v0.36 (parameters, LEADING:20 TRAILING:20 SLIDINGWINDOW:4:20 MINLEN:50 ILLUMINACLIP:NexteraPE-PE.fa:2:30:10) (17). Mitochondrial sequences were discarded by mapping the cleaned reads onto the A. fumigatus mitochondrial genome (reference genome JQ346807) with SMALT v0.7.6. Remaining nuclear reads were assembled with SPAdes v3.7.0 (18) on Calcul Québec servers (http://www.calculquebec.ca/). An in-house Python script (https://github.com/EricFournier3/PythonGenomicTools) was used to eliminate contigs with coverage lower than 5× and length less than 2 kb. The quality of the assembly was further assessed with QUAST 4.1 (19). The absence of duplicated contigs was ensured by running dedup.sh (https://jgi.doe.gov/data-and-tools/bbtools/), and the absence of Illumina adapters was checked with the Linux grep command. A total of 939 contigs for HMR-AF-23 (*N*_50_ = 110,871 bp) and 1,043 contigs for HMR-AF-1038 (*N*_50_ = 95,868 bp) were found. The overall genome size and G+C content for HMR-AF-23 and HMR-AF-1038 are 32,352,689 bp and 48% and 34,076,230 bp and 46%, respectively.

Genome annotation was performed with funannotate v1.4.2 (https://github.com/nextgenusfs/funannotate). We used an Augustus (20) pretrained gene model of A. fumigatus to predict 9,223 genes for HMR-AF-23, including 833 with functional annotation. For HMR-AF-1038, 9,174 genes were predicted, from which 830 have functional annotation. The completeness of these annotations was assessed by comparing the number of conserved orthologs to those of A. fumigatus, the closest reference species, using the tool BUSCO v2.0 (21) run with the Dikarya data set. Approximately 94% orthologs were found for both isolates, which is nearly as many as for A. fumigatus (96%). We found 1,232 and 36 complete and fragmented orthologs, respectively, for HMR-AF-23, compared to 1,227 and 39, respectively, for HMR-AF-1038.

These first draft genome sequences of A. turcosus may be useful in deciphering the genetic differences which result in elevated azole resistance for this species and allow a better understanding of virulence through genomic comparison with other related clinically relevant *Aspergillus* species.

### Data availability.

These whole-genome shotgun projects have been deposited in GenBank under the accession numbers NKHV00000000 for strain HMR-AF-23 and NIDN00000000 for strain HMR-AF-1038. The versions described in this paper are the second versions, NKHV02000000 and NIDN02000000, respectively. Raw sequence data can be accessed using the following run numbers: SRR8165489 (HMR-AF-23) and SRR8165475 (HMR-AF-1038).

## References

[1] Neofytos D, Horn D, Anaissie E, Steinbach W, Olyaei A, Fishman J, Pfaller M, Chang C, Webster K, Marr K 2009 Epidemiology and outcome of invasive fungal infection in adult hematopoietic stem cell transplant recipients: analysis of multicenter Prospective Antifungal Therapy (PATH) Alliance registry. Clin Infect Dis 48:265–273. doi:10.1086/595846. 19115967

[2] Pappas PG, Alexander BD, Andes DR, Hadley S, Kauffman CA, Freifeld A, Anaissie EJ, Brumble LM, Herwaldt L, Ito J, Kontoyiannis DP, Lyon GM, Marr KA, Morrison VA, Park BJ, Patterson TF, Perl TM, Oster RA, Schuster MG, Walker R, Walsh TJ, Wannemuehler KA, Chiller TM 2010 Invasive fungal infections among organ transplant recipients: results of the Transplant-Associated Infection Surveillance Network (TRANSNET). Clin Infect Dis 50:1101–1111. doi:10.1086/651262. 20218876

[3] Kontoyiannis DP, Marr KA, Park BJ, Alexander BD, Anaissie EJ, Walsh TJ, Ito J, Andes DR, Baddley JW, Brown JM, Brumble LM, Freifeld AG, Hadley S, Herwaldt LA, Kauffman CA, Knapp K, Lyon GM, Morrison VA, Papanicolaou G, Patterson TF, Perl TM, Schuster MG, Walker R, Wannemuehler KA, Wingard JR, Chiller TM, Pappas PG 2010 Prospective surveillance for invasive fungal infections in hematopoietic stem cell transplant recipients, 2001–2006: overview of the Transplant-Associated Infection Surveillance Network (TRANSNET) database. Clin Infect Dis 50:1091–1100. doi:10.1086/651263. 20218877

[4] Steinbach WJ, Marr KA, Anaissie EJ, Azie N, Quan SP, Meier-Kriesche H-U, Apewokin S, Horn DL 2012 Clinical epidemiology of 960 patients with invasive aspergillosis from the PATH Alliance registry. J Infect 65:453–464. doi:10.1016/j.jinf.2012.08.003. 22898389

[5] Nierman WC, Pain A, Anderson MJ, Wortman JR, Kim HS, Arroyo J, Berriman M, Abe K, Archer DB, Bermejo C, Bennett J, Bowyer P, Chen D, Collins M, Coulsen R, Davies R, Dyer PS, Farman M, Fedorova N, Fedorova N, Feldblyum TV, Fischer R, Fosker N, Fraser A, García JL, García MJ, Goble A, Goldman GH, Gomi K, Griffith-Jones S, Gwilliam R, Haas B, Haas H, Harris D, Horiuchi H, Huang J, Humphray S, Jiménez J, Keller N, Khouri H, Kitamoto K, Kobayashi T, Konzack S, Kulkarni R, Kumagai T, Lafon A, Lafton A, Latgé J-P, Li W, Lord A, 2005 Genomic sequence of the pathogenic and allergenic filamentous fungus *Aspergillus fumigatus*. Nature 438:1151–1156. doi:10.1038/nature04332. 16372009

[6] Galagan JE, Calvo SE, Cuomo C, Ma L-J, Wortman JR, Batzoglou S, Lee S-I, Baştürkmen M, Spevak CC, Clutterbuck J, Kapitonov V, Jurka J, Scazzocchio C, Farman M, Butler J, Purcell S, Harris S, Braus GH, Draht O, Busch S, D'Enfert C, Bouchier C, Goldman GH, Bell-Pedersen D, Griffiths-Jones S, Doonan JH, Yu J, Vienken K, Pain A, Freitag M, Selker EU, Archer DB, Peñalva MÁ, Oakley BR, Momany M, Tanaka T, Kumagai T, Asai K, Machida M, Nierman WC, Denning DW, Caddick M, Hynes M, Paoletti M, Fischer R, Miller B, Dyer P, Sachs MS, Osmani SA, Birren BW 2005 Sequencing of *Aspergillus nidulans* and comparative analysis with *A. fumigatus* and *A. oryzae*. Nature 438:1105–1115. doi:10.1038/nature04341. 16372000

[7] Machida M, Asai K, Sano M, Tanaka T, Kumagai T, Terai G, Kusumoto K-I, Arima T, Akita O, Kashiwagi Y, Abe K, Gomi K, Horiuchi H, Kitamoto K, Kobayashi T, Takeuchi M, Denning DW, Galagan JE, Nierman WC, Yu J, Archer DB, Bennett JW, Bhatnagar D, Cleveland TE, Fedorova ND, Gotoh O, Horikawa H, Hosoyama A, Ichinomiya M, Igarashi R, Iwashita K, Juvvadi PR, Kato M, Kato Y, Kin T, Kokubun A, Maeda H, Maeyama N, Maruyama J-i, Nagasaki H, Nakajima T, Oda K, Okada K, Paulsen I, Sakamoto K, Sawano T, Takahashi M, Takase K, Terabayashi Y, Wortman JR, Yamada O, Yamagata Y, Anazawa H, Hata Y, Koide Y, Komori T, Koyama Y, Minetoki T, Suharnan S, Tanaka A, Isono K, Kuhara S, Ogasawara N, Kikuchi H 2005 Genome sequencing and analysis of *Aspergillus oryzae*. Nature 438:1157–1161. doi:10.1038/nature04300. 16372010

[8] Samson RA, Hong S, Peterson SW, Frisvad JC, Varga J 2007 Polyphasic taxonomy of *Aspergillus* section *Fumigati* and its teleomorph *Neosartorya*. Stud Mycol 59:147–203. doi:10.3114/sim.2007.59.14. 18490953 PMC2275200

[9] Balajee SA, Gribskov J, Brandt M, Ito J, Fothergill A, Marr KA 2005 Mistaken identity: *Neosartorya pseudofischeri* and its anamorph masquerading as *Aspergillus fumigatus*. J Clin Microbiol 43:5996–5999. doi:10.1128/JCM.43.12.5996-5999.2005. 16333088 PMC1317194

[10] Balajee SA, Nickle D, Varga J, Marr KA 2006 Molecular studies reveal frequent misidentification of *Aspergillus fumigatus* by morphotyping. Eukaryot Cell 5:1705–1712. doi:10.1128/EC.00162-06. 17030996 PMC1595351

[11] Balajee SA, Kano R, Baddley JW, Moser SA, Marr KA, Alexander BD, Andes D, Kontoyiannis DP, Perrone G, Peterson S, Brandt ME, Pappas PG, Chiller T 2009 Molecular identification of *Aspergillus* species collected for the Transplant-Associated Infection Surveillance Network. J Clin Microbiol 47:3138–3141. doi:10.1128/JCM.01070-09. 19675215 PMC2756904

[12] Katz ME, Dougall AM, Weeks K, Cheetham BF 2005 Multiple genetically distinct groups revealed among clinical isolates identified as atypical *Aspergillus fumigatus*. J Clin Microbiol 43:551–555. doi:10.1128/JCM.43.2.551-555.2005. 15695644 PMC548029

[13] Hong S-B, Shin H-D, Hong J, Frisvad JC, Nielsen PV, Varga J, Samson RA 2008 New taxa of *Neosartorya* and *Aspergillus* in *Aspergillus* section *Fumigati*. Antonie Van Leeuwenhoek 93:87–98. doi:10.1007/s10482-007-9183-1. 17610141 PMC2140094

[14] Samson RA, Visagie CM, Houbraken J, Hong SB, Hubka V, Klaassen CHW, Perrone G, Seifert KA, Susca A, Tanney JB, Varga J, Kocsubé S, Szigeti G, Yaguchi T, Frisvad JC 2014 Phylogeny, identification and nomenclature of the genus *Aspergillus*. Stud Mycol 78:141–173. doi:10.1016/j.simyco.2014.07.004. 25492982 PMC4260807

[15] CLSI. 2008 Reference method for broth dilution antifungal susceptibility testing of filamentous fungi; approved standard, 2nd ed CLSI Document MM38-A2. Clinical and Laboratory Standards Institute, Wayne, PA.

[16] Espinel-Ingroff A, Diekema DJ, Fothergill A, Johnson E, Pelaez T, Pfaller MA, Rinaldi MG, Canton E, Turnidge J 2010 Wild-type MIC distributions and epidemiological cutoff values for the triazoles and six *Aspergillus* spp. for the CLSI broth microdilution method (M38-A2 document). J Clin Microbiol 48:3251–3257. doi:10.1128/JCM.00536-10. 20592159 PMC2937688

[17] Bolger AM, Lohse M, Usadel B 2014 Trimmomatic: a flexible trimmer for Illumina sequence data. Bioinformatics 30:2114–2120. doi:10.1093/bioinformatics/btu170. 24695404 PMC4103590

[18] Nurk S, Bankevich A, Antipov D, Gurevich A, Korobeynikov A, Lapidus A, Prjibelsky A, Pyshkin A, Sirotkin A, Sirotkin Y, Stepanauskas R, McLean J, Lasken R, Clingenpeel SR, Woyke T, Tesler G, Alekseyev MA, Pevzner PA 2013 Assembling genomes and mini-metagenomes from highly chimeric reads, p 158–170. *In* Deng M, Jiang R, Sun F, Zhang X (ed), Research in computational molecular biology: 17th annual international conference, RECOMB 2013, Beijing, China, April 7–10, 2013 proceedings. Springer, Berlin, Heidelberg. doi:10.1007/978-3-642-37195-0_13.

[19] Gurevich A, Saveliev V, Vyahhi N, Tesler G 2013 QUAST: quality assessment tool for genome assemblies. Bioinformatics 29:1072–1075. doi:10.1093/bioinformatics/btt086. 23422339 PMC3624806

[20] Stanke M, Schöffmann O, Morgenstern B, Waack S 2006 Gene prediction in eukaryotes with a generalized hidden Markov model that uses hints from external sources. BMC Bioinformatics 7:62. doi:10.1186/1471-2105-7-62. 16469098 PMC1409804

[21] Simão FA, Waterhouse RM, Ioannidis P, Kriventseva EV, Zdobnov EM 2015 BUSCO: assessing genome assembly and annotation completeness with single-copy orthologs. Bioinformatics 31:3210–3212. doi:10.1093/bioinformatics/btv351. 26059717

